# Graph-based deep learning for integrating single-cell and bulk transcriptomic data to identify clinical cancer subtypes

**DOI:** 10.1093/bib/bbaf467

**Published:** 2025-09-11

**Authors:** Yixin Liu, Dandan Zhang, Tianyu Liu, Ao Wang, Guohua Wang, Yuming Zhao

**Affiliations:** Modern Education Technology Center, Harbin Medical University, 157 Baojian Road, Nangang District, Harbin 150080, China; Department of Obstetrics and Gynecology, The First Affiliated Hospital of Harbin Medical University, 199 Dazhi Street, Nangang District, Harbin 150007, China; College of Computer and Control Engineering, Northeast Forestry University, 26 Hexing Road, Xiangfang District, Harbin 150040, China; College of Computer and Control Engineering, Northeast Forestry University, 26 Hexing Road, Xiangfang District, Harbin 150040, China; College of Computer and Control Engineering, Northeast Forestry University, 26 Hexing Road, Xiangfang District, Harbin 150040, China; College of Computer and Control Engineering, Northeast Forestry University, 26 Hexing Road, Xiangfang District, Harbin 150040, China

**Keywords:** single-cell RNA sequencing, data integration, cancer subtypes, graph-based deep learning, prediction model

## Abstract

The integration of single-cell RNA sequencing (scRNA-seq) and bulk transcriptomic data has become essential for deciphering the complex heterogeneity of cancer and identifying clinical cancer subtypes. However, the inherent challenges posed by the high dimensionality, sparsity, and noise characteristics of scRNA-seq data have significantly hindered its widespread clinical translation. To address these limitations, we introduce single-cell and bulk transcriptomic graph deep learning, a graph-based deep learning method that synergistically integrates scRNA-seq and bulk transcriptomic data to precisely identify cancer subtypes and predict clinical outcomes. scBGDL constructs sample-specific gene graphs modeling complex gene–gene interactions and cellular relationships. The architecture employs Graph Attention Networks for feature aggregation, MinCutPool layers for dimensionality reduction, and Transformer modules to capture high-order biological dependencies. Independently validated in each of 16 distinct The Cancer Genome Atlas cancer types, scBGDL significantly outperformed existing methods in prognostic accuracy (mean C-index: 0.7060 versus 0.6709 max competitor), demonstrating robustness and generalizability to diverse transcriptional architectures. To demonstrate clinical versatility, we further evaluated scBGDL in three therapeutic contexts using multicenter cohorts: lung adenocarcinoma survival prediction (*n* = 1099), epithelial ovarian cancer platinum-based chemotherapy response (*n* = 762), skin cutaneous melanoma immunotherapy outcome (*n* = 305). scBGDL consistently delivered robust risk stratification (log-rank *P* < 0.05 across cohorts), identified key driver edges, and uncovered clinically relevant biological interpretations. By enabling multimodal data integration and interpretable biological insights, scBGDL advances precision oncology for prognosis prediction, therapy optimization, and biomarker discovery. The source code for scBGDL model is available online (https://github.com/NEFLab/scBGDL).

## Introduction

Single-cell RNA sequencing (scRNA-seq) has revolutionized our understanding of cancer biology by enabling the precise characterization of cellular heterogeneity within tumors and their microenvironments at single-cell resolution [1]. It identifies distinct cancer subpopulations, uncovers cell–cell interactions, and provides insights into tumor biology, driving the discovery of therapeutic targets and advancing precision medicine [2, 3]. Nevertheless, scRNA-seq's high cost, technical complexity, and limited scalability to large cohorts often result in small sample sizes, limiting statistical power to link findings with clinical outcomes like survival and treatment response. These constraints hinder the translation of scRNA-seq findings into diagnostic and therapeutic advancements [4].

Integrating scRNA-seq with bulk transcriptomic has emerged as a promising strategy to bridge single-cell biology with population-level clinical insights [5–8]. By leveraging the complementary strengths of these two data modalities, researchers can overcome the limitations of small single-cell datasets while exploring the clinical relevance of cellular heterogeneity [9]. This integration facilitates more comprehensive analyses of cancer subtypes and enables the development of more precise prognostic and therapeutic models. However, current integration methods face critical challenges. Feature-based fusion and multiresolution techniques often fail to adequately address scRNA-seq sparsity and noise, leading to information loss and compromised predictive accuracy [10, 11]. Moreover, few models robustly capture higher-order gene–gene interactions essential for clinical outcome prediction [12].

Here, we present scBGDL (Single-Cell and Bulk Transcriptomic Graph Deep Learning), a graph neural network framework designed to overcome these limitations. scBGDL innovates by: (i) Constructing sample-specific gene graphs where nodes represent clinically informed key genes and edges encode expression-derived relationships, (ii) Implementing a multitiered architecture combining graph attention network layers (for adaptive neighbor aggregation), MinCutPool (for hierarchical graph compression), and Transformers (for global context modeling) [13, 14], (iii) Incorporating a regularized Cox loss to optimize survival prediction while preserving biological interpretability. Our validation follows a two-phase framework: For technical robustness, we rigorously validated scBGDL across 16 cancer genome atlas (CGA) cancer types, demonstrating superior prognostic performance versus existing methods to diverse transcriptomic architectures. To establish clinical utility, we further assessed scBGDL across diverse therapeutic architectures using multicenter cohorts: stratifying lung adenocarcinoma (LUAD) survival risk, predicting epithelial ovarian cancer (EOC) platinum chemotherapy resistance, and identifying skin cutaneous melanoma (SKCM) immunotherapy responders. Beyond predictive accuracy, scBGDL extracts biological interpretations through node-edge-group analysis, revealing key driver edges and dysregulated pathways. This positions scBGDL as a bridge between computational biology and clinical practice, uniquely integrating these fields to provide a scalable and innovative computational paradigm that advances the goals of precision oncology.

## Materials and methods

### Development of scBGDL

**Figure 1:**
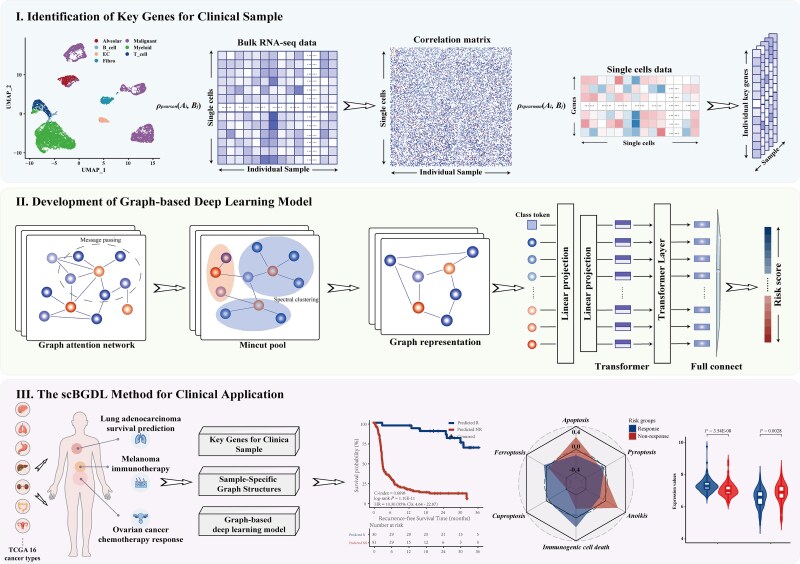
Schematic illustration of scBGDL for integrating single-cell and bulk transcriptomic data to identify clinical cancer subtypes.

The scBGDL integrates single-cell and bulk RNA-seq data to construct sample-specific gene graphs and leverages graph-based deep learning with self-attention mechanisms for precise cancer subtype classification and survival prediction. As illustrated in Fig. 1, the workflow comprises four integrated phases:

#### Phase 1: identification of clinically relevant key genes

##### Survival-associated gene screening

Conducted univariate Cox regression on bulk transcriptomic to evaluate gene expression associations with overall survival (OS). Aligned gene sets across single-cell and bulk modalities to generate a standardized multimodal expression matrix. Computed Spearman’s rank correlation (*Rho*) between single-cell and bulk samples, generating correlation coefficients (*Rho*) and adjusted *P* values using false discovery rate (FDR) control.

##### CorGene score calculation

Following a previously described approach [15], we defined a *CorGene* score to quantify the magnitude and statistical significance of correlations, as defined in (1):


(1) \begin{equation*} {CorGene}_{(i)}=\left\{\begin{array}{@{}c}\ 1-2\times FDR; if\ Rho>0\ \\{}2\times FDR-1; if\ Rho<0\end{array}\right. \end{equation*}

Genes with absolute *CorGene* scores >0.995 and FDR < 0.05 were deemed significant and assigned as key genes for each sample.

#### Phase 2: construction of sample-specific gene graphs

##### Graph definition

In the sample-specific graph, nodes represent key genes, with node features derived from gene expression values, and edges are determined based on gene expression similarity.

##### Edge construction

To define the connectivity between nodes, an adjacency matrix for single-cell and bulk data is constructed to represent gene relationships using Uniform Manifold Approximation and Projection (UMAP) based pairwise similarity. For each gene, its *k*-nearest neighbors were identified to construct a binary adjacency matrix, where edges represent connections between sufficiently close genes. The adjacency matrices derived from single-cell data and bulk transcriptomic data are element-wise summed to generate a fused adjacency matrix (*A_Matrix_*), as defined in (2):


(2) \begin{equation*} {A}_{Matrix}\left[i,j\right]=\left\{\begin{array}{@{}c}\ 1; if\ {A}_{Matrix}\left[i,j\right]>0\ \\{}0; if\ {A}_{Matrix}\left[i,j\right]=0\end{array}\right. \end{equation*}

The fused matrix was binarized, such that if any gene pair was connected in either modality, the connection was retained in the final adjacency matrix.

#### Phase 3: graph neural network architecture

Each clinical sample is represented as a graph whose nodes denote key genes. Edges represent expression-similarity relationships.

##### Graph attention network (GAT)

For each node ${\nu}_i$, its feature vector ${h}_i\in{\mathbb{R}}^F$ is updated based on its neighboring nodes. The GAT computes normalized attention coefficients that quantify the importance of edges. The attention mechanism is defined in (3):


(3) \begin{equation*} {e}_{ij}=f\left({a}^T\left[W{h}_i\parallel W{h}_j\right]\right) \end{equation*}

where $W\in{\mathbb{R}}^{F\times{F}^{\prime }}$ is a shared weight matrix, $f\left(\cdot \right)$ is a feed-forward network, and $\parallel$ denotes vector concatenation. Applying the softmax function over the neighborhood ${\mathcal{N}}_i$ of node *i* yields normalized attention coefficients ${a}_{ij}$, as defined in (4):


(4) \begin{equation*} {a}_{ij}=\frac{\mathit{\exp}\left(f\left({e}_{ij}\right)\right)}{\sum_{k\in{\mathcal{N}}_i}\mathit{\exp}\left({e}_{ik}\right)} \end{equation*}

These coefficients are used to aggregate features from neighboring nodes, as defined in (5):


(5) \begin{equation*} {h}_i^{\prime }=\sigma \left(\sum_{j\in{\mathcal{N}}_i}{a}_{ij}W{h}_j\right) \end{equation*}

where $\sigma \left(\cdot \right)$ denotes a nonlinear activation function, such as LeakyReLU.

##### MinCut pooling

Applied MinCutPool to cluster nodes hierarchically, reducing graph complexity while preserving critical topological features.

##### Transformer module

Applied Transformer module to capture global context and high-order relationships. Each node is treated as a token, and a class token is added to represent global properties. The Transformer module applies multihead self-attention and feed-forward networks, as defined in (6) and (7):


(6) \begin{equation*} {Z}_l^{\prime }= LN\left({Z}_{l-1}+ MSA\left({Z}_{l-1}\right)\right) \end{equation*}

(7) \begin{equation*} {Z}_l= LN\left({Z}_l^{\prime }+ FFN\left({Z}_l^{\prime}\right)\right) \end{equation*}

where ${Z}_{l-1}$ and ${Z}_l$ are the inputs and outputs of the *l*-th layer, respectively. Positional encodings and encodings derived from the adjacency matrix are also incorporated to introduce structural information.

##### Survival prediction

Survival prediction was framed as a proportional hazards problem using the Cox partial log-likelihood as the primary loss function, as defined in (8):


(8) \begin{equation*} {\mathcal{L}}_{Cox}=-\sum_{i\in E}\left({\theta}_i-\mathit{\log}\sum_{j\in{\mathcal{R}}_i}{e}^{\theta_j}\right) \end{equation*}

where *E* is the set of uncensored patients, and ${\mathcal{R}}_i$ is the set of patients with shorter observed survival times than patient *i*. To prevent degenerate graph pooling solutions and maintain meaningful graph structures, we incorporated a MinCutPool regularization term ${\mathcal{L}}_{pool}$, leading to the final objective function, as defined in (9):


(9) \begin{equation*} \mathcal{L}={\mathcal{L}}_{Cox}+\lambda{\mathcal{L}}_{pool} \end{equation*}

where $\lambda$ balances the contributions of survival prediction and graph regularization.

##### Implementation details

To avoid overfitting, we applied early stopping during training. If the improvement in the loss function fell below a predefined threshold over several epochs, training was terminated. The trained model outputs a risk score for each patient, which can be converted into survival probabilities. The optimal cut-off for risk scores was determined using the “survminer” R package, refining the predictive power of the model. This model was implemented in PyTorch and trained on NVIDIA RTX 4090 GPU.

#### Phase 4: model interpretability analysis

To enhance the clinical plausibility of scBGDL and ensure a robust biological basis, we employed an integration analysis of node-edge-group differences to deconstruct the decision mechanism of graph attention networks.

##### Node-level analysis

Integrated gene node importance across global, high-risk, and low-risk cohorts to compute risk bias, logarithmic fold-change, and node centrality. Key genes are identified through tri-criteria screening (global ranking, differential significance, centrality) and classified by risk bias polarity.

##### Edge-level analysis

Edge weight data are integrated to quantify risk specificity and edge influence, filtering interactions connecting key genes for functional categorization.

##### Driver mechanism extraction

Louvain algorithm detects functional modules, with characteristics quantified through topological properties, risk composition, and interaction strength. Multidimensional integration ultimately prioritizes cross-group edges connecting opposing-risk nodes, revealing their role as core regulatory hubs in cancer risk stratification.

### Experiment datasets

Pan-cancer bulk RNA-seq datasets were obtained from TCGA, http://cancergenome.nih.gov/). Inclusion criteria comprised: (i) patients with complete 5-year survival follow-up records; (ii) cancer types containing ≥150 uncensored survival events. In total, 6893 samples across 16 cancer types met these criteria, with cohort sizes ranging from 159 to 1081 patients per cancer type. Complementary pan-cancer scRNA-Seq data were acquired from TISCH portal (http://tisch.comp-genomics.org/), encompassing 137 patients and 701 031 cells. These span 16 malignancies: bladder urothelial carcinoma, glioma, breast cancer (BRCA), colorectal cancer (CRC), esophageal squamous cell carcinoma (ESCA), head and neck squamous carcinoma (HNSC), kidney renal clear cell carcinoma, liver hepatocellular carcinoma, non-small cell lung cancer, pancreatic adenocarcinoma (PAAD), cervical squamous cell carcinoma and endocervical adenocarcinoma, ovarian serous cystadenocarcinoma (OV), uterine corpus endometrial carcinoma (UCEC), prostate adenocarcinoma, SKCM, and stomach adenocarcinoma. These details of the analyzed datasets are displayed in Table S1.

To evaluate clinical utility across diverse therapeutic contexts, we further assessed scBGDL in diverse oncology contexts using multicenter cohorts: LUAD survival prediction (Table S2), EOC platinum-based chemotherapy response (Table S3), SKCM immunotherapy outcome (Table S4).

#### Lung adenocarcinoma datasets (survival prediction)

The LUAD scRNA-seq dataset was obtained from the ArrayExpress repository (accession code: E-MTAB-6149), encompassing 40 218 cells categorized into 12 distinct cell types [16]. Bulk RNA-seq data of 1099 LUAD patients were retrieved from six public datasets via Gene Expression Omnibus (GEO) and TCGA. The largest dataset (GSE68465, *n* = 333) served as discovery cohort. Validation cohorts consisted of TCGA-LUAD (*n* = 267), GSE31210 (*n* = 204), GSE50081 (*n* = 127), GSE30219 (*n* = 81), and GSE42127 (*n* = 87).

#### Epithelial ovarian cancer datasets (chemotherapy response)

The EOC scRNA-seq dataset was retrieved from GEO (accession code: GSE158722), comprising 96,846 cells grouped into four distinct cell types [17]. Bulk RNA-seq data from 762 EOC patients were obtained from three public datasets available via GEO and TCGA. The largest dataset (TCGA-OV, *n* = 377) served as discovery cohort. Validation cohorts consisted of GSE9891 (*n* = 278) and GSE26193 (*n* = 107).

#### Skin cutaneous melanoma datasets (immunotherapy outcome)

The SKCM scRNA-seq dataset was obtained from GEO (accession code: GSE159251), comprising 95,421 cells categorized into four distinct cell types [18]. Bulk RNA-seq data for 305 SKCM patients were retrieved from five independent public datasets. The discovery cohort, consisting of 121 patients with recurrence-free survival (RFS) data, was obtained from Liu’s study [19]. The RFS-validation cohort was integrated from Gide’s study [20] (*n* = 73) and Van Allen’s study [21] (*n* = 34). The OS-validation cohort were compiled from Hugo’s study [22] (*n* = 26) and Riaz’s study [23] (*n* = 51).

For the preprocessing, scRNA-seq data were processed using Seurat (v5.0.2): Low-expression genes were filtered, and expression matrices were normalized via ‘NormalizeData’. Microarray data underwent RMA normalization; RNA-seq data were FPKM-normalized and log₂-transformed. Ensemble gene IDs were converted to Entrez IDs for standardization. Batch effects were corrected using ComBat method from the ``sva'' R package.

### Comparison methods

scBGDL was rigorously compared to three established integration methodologies. A brief overview of these methods follows:

Scissor [5]: identifies cell subpopulations in scRNA-seq data that are closely linked to specific phenotypes. Using a sparse regression model, it pinpoints key genes shaping the expression profiles of these subpopulations.

scAB [6]: detects clinically significant cell states across multiple resolutions through a knowledge- and graph-guided matrix factorization framework. By integrating multiresolution cell states and hierarchical structures, it offers insights into critical cell states associated with phenotypes.

LP_SGL [8]: inspired by the analogy between community connectivity in networks and cell–cell interactions in scRNA-seq data, applies the Leiden algorithm for community detection. It identifies cell groups based on their interactions in a graph structure and links these groups with bulk expression and phenotype data to uncover subpopulations associated with particular clinical phenotypes.

### Evaluation metrics

Overall survival was defined as the time from initial surgical resection to death or last contact, while RFS was defined as the time to disease progression or death. Both metrics were truncated at 60 and 36 months to account for varying follow-up times. Kaplan–Meier survival curves were estimated and compared using the log-rank test [24]. Cox regression models (univariate and multivariate) were used to assess clinical factors' association with OS and determine the prognostic value of identified factors. Hazard ratios (HRs) with 95% confidence intervals (CIs) and the C-index [25] were calculated to evaluate survival predictors.

Fisher’s exact test was applied to assess the statistical significance of overlaps and associations among various groups. Functional enrichment analyses were conducted using the ‘clusterProfiler’ package in R, referencing the Kyoto Encyclopedia of Genes and Genomes (KEGG) database. Student’s t-tests were used to compare differences in molecular scores derived from mRNA expression profiles across groups. Molecular signatures, including scores of hypoxia [26], stemness [27], proliferation [28], and immune [29], as well as regulatory cell death patterns, diverse immune cell types within the tumor microenvironment, LUAD driver genes, and immune stimulatory genes, were assessed. Additional details are provided in the Supplementary Methods. The chi-square test was used to examine the association of response groups predicted by the identified model with known molecular subtypes of EOC.

All statistical analyses were performed in R version 4.3.3. Statistical significance was defined as two-sided *P* < 0.05 or FDR < 0.05 for multiple testing.

## Results

The scBGDL method for clinical application in cancer representation learning.

The scBGDL method integrates single-cell and bulk transcriptomic data to construct sample-specific gene-graph networks for cancer subtype prediction and survival analysis. By aligning single-cell and bulk RNA-seq datasets, a standardized multimodal expression matrix is generated to identify significant genes for each sample. Sample-specific graphs are then built, with nodes representing key genes and edges encoding expression-based similarities. The graphs are processed by a graph-based deep learning model combining GATs for feature aggregation, MinCutPool layers for complexity reduction, and a Transformer module for capturing higher-order gene relationships. The model is trained using a Cox partial log-likelihood loss function to optimize survival prediction while maintaining graph sparsity. scBGDL delivers robust survival predictions and identifies pathways and mechanisms associated with cancer subtypes.

**Figure 2:**
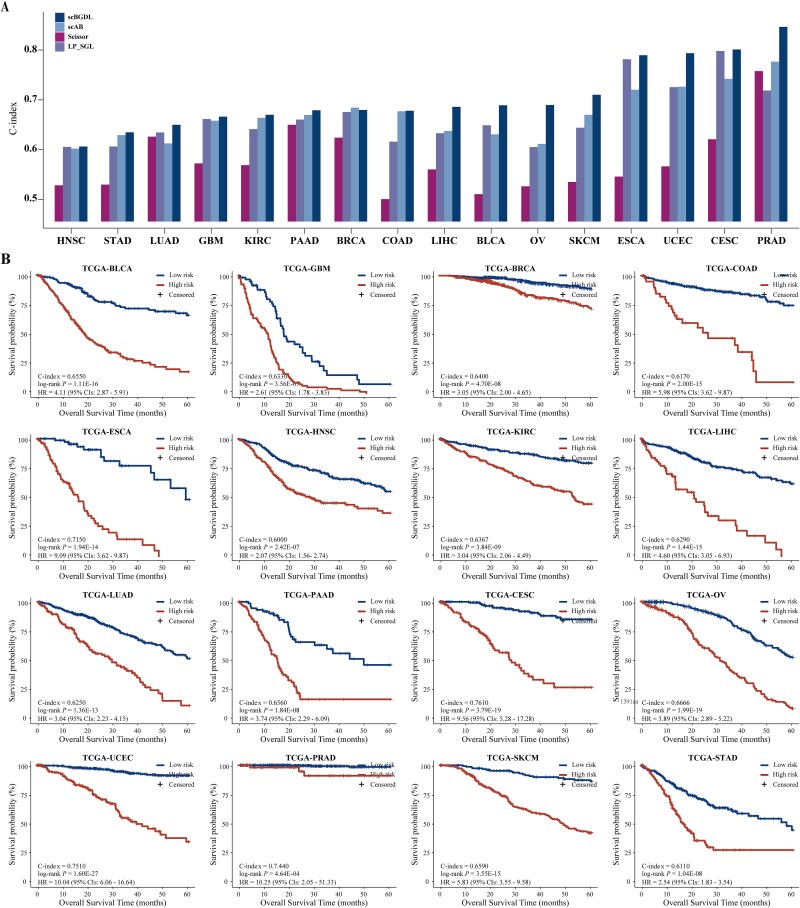
Performance comparison of scBGDL and baseline methods in prognosis prediction. (A) Comparisons of the C-indexes of Scissor, scAB, LP_SGL, and scBGDL models in each cancer type. (B) Kaplan–Meier analysis of patients stratified into high- and low-risk groups based on scBGDL model across 16 cancer types, statistical significances were assessed using the log-rank test (*P* < .05).

We initially assessed the technical robustness of scBGDL in predicting cancer patient prognosis on 16 TCGA cancer types. (Fig. 2A, Table S5), utilizing single-cell and bulk transcriptomic data as input. We compared scBGDL against other major competitors, including Scissor, scAB, and LP_SGL, under the same data preprocessing and parameter settings as originally described in their respective publications. scBGDL demonstrated the highest overall average C-index across 16 cancer types at 0.7060 (Table S5). In contrast, the average C-index of Scissor, scAB, and LP_SGL was 0.5781, 0.6709, and 0.6670, respectively. Although scAB achieved a slightly higher C-index than scBGDL in BRCA (scAB: 0.6855 versus scBGDL: 0.6809), scBGDL exhibited the highest C-index performance in all other cancers. Overall, scBGDL achieved an average improvement of 22.12%, 5.23%, and 5.85% compared to the collective performance of the other three methods. For instance, in ESCA and UCEC, scBGDL outperformed alternatives by >40% versus Scissor and >9% versus scAB, underscoring its robustness in high-mortality cancers. We further employed Kaplan–Meier survival curves to visually assess the stratification quality of model-predicted high-risk and low-risk patient groups. Log-rank tests were conducted to evaluate statistical differences in survival outcomes between these groups. scBGDL achieved statistically significant risk stratification (log-rank *P* < .05, Fig. 2B) across 16 cancer types, highlighting its robustness in clinical prognosis prediction. These results validate scBGDL as a superior method for integrating multi-omics data to refine prognostic accuracy.

### Identifying clinical subtypes associated with LUAD prognosis

**Figure 3:**
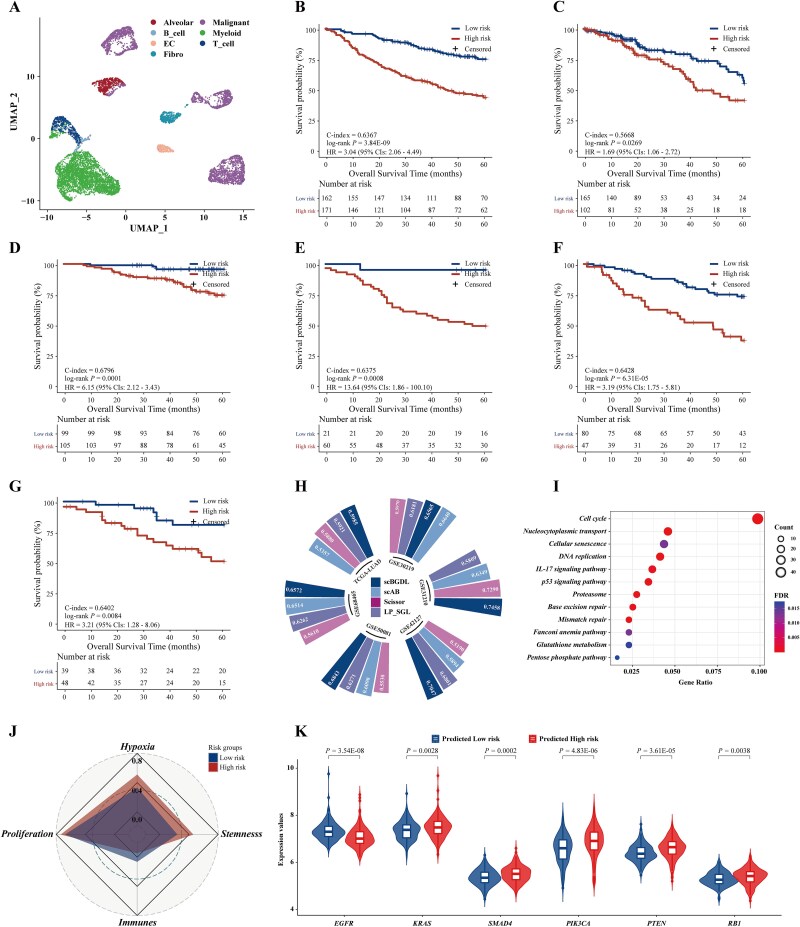
The scBGDL method for identifying clinical prognostic subtypes in lung adenocarcinoma. (A) UMAP visualization of 8564 LUAD cancer cells grouped into seven clusters. (B) Kaplan–Meier curves showing overall survival (OS) for 333 patients in GSE68465 discovery cohort. (C–G) Kaplan–Meier curves for OS in validation cohorts, including TCGA-LUAD (*n* = 267), GSE31210 (*n* = 204), GSE50081 (*n* = 127), GSE30219 (*n* = 81), and GSE42127 (*n* = 87). (H) Comparison of biomarker performance derived from different methods using C-index. (I) Kyoto Encyclopedia of genes and genomes (KEGG) functional enrichment analysis of 794 genes distinguishing high-risk and low-risk groups in GSE68465 cohort. (J) A radar chart displaying hypoxia, proliferation, stemness, and immune scores between two risk groups. (K) Split violin plots showing significant differences in six LUAD driver genes between the two risk groups. Statistical differences were calculated using Student’s t-test.

To evaluate clinical utility for prognostic assessment, we applied scBGDL to LUAD datasets. From GSE68465 discovery cohort, we identified a set of 2343 prognostic genes (*P* < .05). After data preprocessing, 8564 cells expressing at least 400 genes were retained from an original set of 40 218 cells spanning various cell types (Fig. 3A). Thereafter, scBGDL identified a set of key genes for each LUAD patient and constructed a graph-structured prognostic model. After optimizing the GDL model using the negative Cox partial log-likelihood loss, we derived risk scores for LUAD patients in GSE68465 cohort. An optimal cut-off (0.1822) classified patients into high-risk and low-risk groups, with high-risk patients exhibiting significantly worse OS (log-rank *P* = 3.84E-09, C-index = 0.6367; Fig. 3B). Using this cut-off, survival analyses across independent validation cohorts consistently showed poorer OS for high-risk patients (Fig. 3C–G): TCGA-LUAD (log-rank *P* = .0269, C-index = 0.5668), GSE31210 (log-rank *P* = .0001, C-index = 0.6796), GSE30219 (log-rank *P* = .0008, C-index = 0.6375), GSE50081 (log-rank *P* = 6.31E-05, C-index = 0.6428), and GSE42127 (log-rank *P* = .0084, C-index = 0.6402). Multivariate Cox analyses confirmed that scBGDL predicted 5-year survival independently of clinical factors in both discovery and validation cohorts (Table S6). We further compared scBGDL against other major competitors, including Scissor, scAB, and LP_SGL, and traditional clinicopathological risk models. Survival analyses confirmed scBGDL's superior performance (Fig. 3H and Table S7). The highest C-index values were observed in LUAD discovery (0.6572) and validation cohorts (TCGA-LUAD, 0.5985; GSE31210, 0.7458; GSE50081, 0.6843; GSE42127, 0.7047), with the exception of GSE30219 cohort.

To enhance the interpretability of the scBGDL, we analyzed node-edge-group differences to construct LUAD prognostic network (Fig. S1A), including 39 high-risk and 172 low-risk core genes connected by 778 edges, and identified 16 key high-risk driver edges (risk specificity >0.5; frequency >20%, Fig. S1B). Among the 27 node genes of these edges, 10/27 genes literature-validated associated with LUAD patients prognosis (e.g. *DNM1L, PNO1, VDAC2, PSMC4, PPAT, GOLT1B, MRPL35, PYGL*). KEGG pathway analysis of 794 DEGs (Student’s t-test, FDR < 0.05) revealed 12 pathways linked to LUAD prognosis, such as “cell cycle”, “DNA replication”, and “p53 signaling pathway” (Hypergeometric test, FDR < 0.05; Fig. 3I). Additionally, High-risk patients had significantly higher scores in hypoxia (*P* = 1.19E-16), proliferation (*P* = 2.88E-51), and stemness (*P* = 2.91E-34), but lower immune scores (Fig. 3J). Analysis of 24 known LUAD driver genes (Table S8) showed high-risk patients had elevated expression of *KRAS* [30] (*P* = .0028), *PIK3CA* [31] (*P* = 4.83E-06), and *PTEN* [32] (*P* = 3.61E-05), while low-risk patients had increased expression of *EGFR* [33] (*P* = 3.54E-08), *SMAD4* (*P* = .0002), and *RB1* [34] (*P* = .0038). These results suggest that genes identified by scBGDL may serve as biomarkers for early diagnosis, screening, and personalized treatment of LUAD.

### Decoding chemotherapy-response subtypes in epithelial ovarian cancer

**Figure 4:**
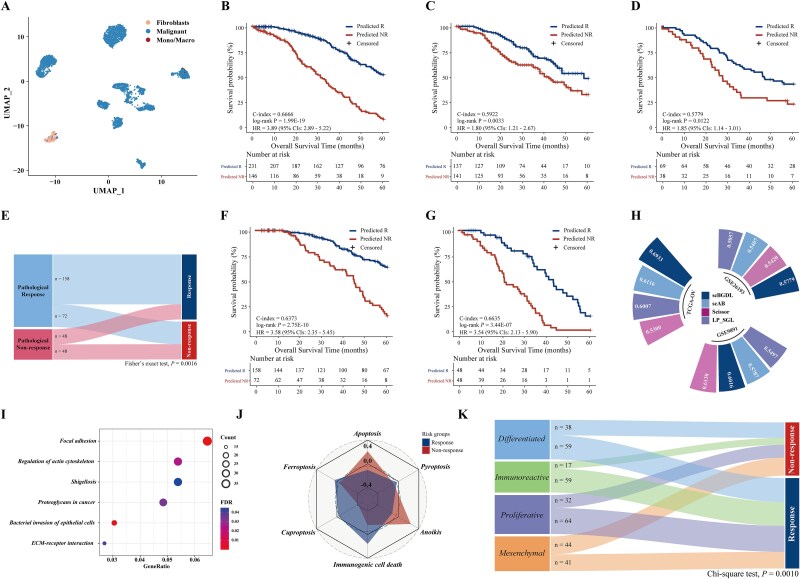
The scBGDL method for identifying sensitivity subtypes to platinum-based adjuvant chemotherapy in epithelial ovarian cancer. (A) UMAP visualization of 3652 EOC cancer cells categorized into three clusters. (B) Kaplan–Meier curves showing OS for 377 patients in TCGA-OV discovery cohort. (C-D) Kaplan–Meier curves for OS in validation cohorts, including GSE9891 (*n* = 278) and GSE26193 (*n* = 107). (E) Sankey diagram illustrating the association between ACT response model predictions and pathological response states. Fisher’s exact test was used to evaluate the association. (F) Kaplan–Meier curves of OS for 230 EOC patients receiving ACT in pathological response group. (G) Kaplan–Meier curves of OS for 96 EOC patients receiving ACT in pathological nonresponse group. (H) Comparison of biomarker performance across different methods using C-index. (I) KEGG functional enrichment analysis of 1296 genes distinguishing nonresponse and response groups in TCGA-OV cohort. (J) A radar chart displaying apoptosis, anoikis, cuproptosis, immunogenic cell death, hypoxia, ferroptosis, and pyroptosis scores between two risk groups. (K) Sankey diagram showing the association between ACT response model predictions and EOC molecular subtypes. Chi-square test was used to assess the relationship.

To further showcase the clinical utility and robustness of scBGDL, we extended its application to evaluating chemotherapy response in EOC patients treated with platinum-based ACT. We focused on 1088 prognostic genes identified in TCGA-OV discovery cohort. From a total of 96 846 cells, we extracted 3652 high-quality single cells expressing ≥400 genes (Fig. 4A). These cells were integrated with bulk RNA-seq data to construct comprehensive molecular profiles for each clinical sample. Using these key genes, we developed a graph-based ACT response model. An optimal cut-off (0.5819) was applied to classify patients into nonresponders and responders. Survival analysis demonstrated a significant difference in OS (log-rank *P* = 1.99E-19, C-index = 0.6666, Fig. 4B) in TCGA-OV cohort. Validation across two independent cohorts confirmed the model's robustness: nonresponders showed shorter OS in GSE9891 (log-rank *P* = .0033, C-index = 0.5922, Fig. 4C) and GSE26193 (log-rank *P* = .0122, C-index = 0.5779, Fig. 4D) cohorts. Multivariate Cox analysis validated the model as an independent predictor of 5-year survival (Table S9). Furthermore, pathological response data from TCGA-OV (230 responders, 96 nonresponders) further highlighted its predictive value, as predicted nonresponders were significantly enriched in pathological nonresponse group (Fisher’s exact test, *P* = .0016; Fig. 4E). Notably, among the 230 pathological complete responders, 72 (31.3%) were reclassified as nonresponders by scBGDL model. Conversely, among the 96 pathological nonresponders, 48 (50.0%) were reclassified as responders. Reclassified nonresponders had significantly shorter OS compared to consistent responders (log-rank *P* = 2.75E-10, C-index = 0.6373, Fig. 4F), while reclassified responders in the pathological nonresponse group showed improved OS (log-rank *P* = 3.44E-07, C-index = 0.6635, Fig. 4G). These findings underscore the model's ability to refine pathological response classifications and improve prognostic predictions in EOC patients. To evaluate the robustness of scBGDL in predicting chemotherapy sensitivity, we compared it with other methods and traditional clinical features. Figure 4H showed that scBGDL demonstrated superior prognostic accuracy, achieving a C-index of 0.6933 in discovery cohort, and C-index values of 0.6016 and 0.5779 in validation cohorts (GSE9891 and GSE26193, Table S10).

To explore associated biological processes, we conducted an integrated analysis to construct EOC ACT response network (Fig. S2A), including 30 high-risk and 62 low-risk core genes connected by 330 edges, and identified 32 key nonresponse driver edges (risk specificity >0.5; frequency >20%, Fig. S2B). Among the 33 node genes of top 20 key driver edges, nine genes were literature-validated associated with EOC patients treated with platinum-based ACT (e.g. *SLC7A11, CMTM4, TRIM27, PROM2, TYMS, CLIC3*). KEGG pathway analysis of 1296 DEGs (Student’s t-test, FDR < 0.05) revealed seven pathways (Hypergeometric test, FDR < 0.05, Fig. 4I), including such as “Focal adhesion”, “Ribosome”, and “ECM-receptor”, associated with platinum-based ACT. Furthermore, the predictive responders exhibited significantly higher scores in apoptosis (*P* = .0084) and anoikis (*P* = .0122), and lower scores in cuproptosis (*P* = .0099) and immunogenic cell death (*P* = .0014), suggesting that cell death regulation plays a critical role in treatment outcomes (Fig. 4J). Meanwhile, we also examined the relationship between response groups and four molecular EOC subtypes defined by TCGA-OV dataset [35], and found that nonresponders were more likely to belong to mesenchymal subtype compared to other subtypes [36] (Chi-square test, *P* = .0010, Fig. 4K), providing biological evidence that our model accurately identifies nonresponders to platinum-based ACT.

### Capturing biomarkers with predictive power for immunotherapy in melanoma

**Figure 5:**
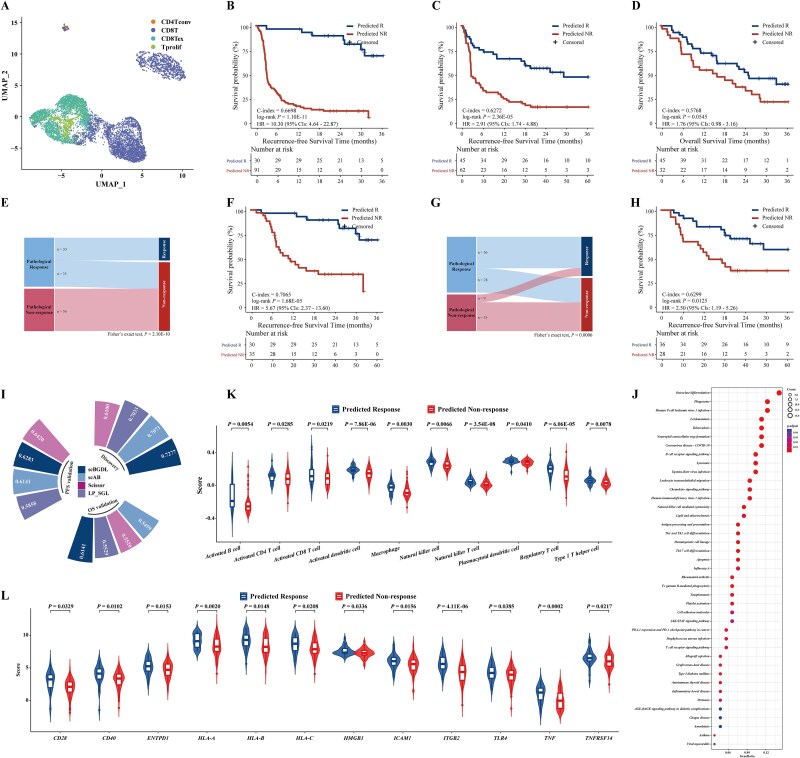
The scBGDL method for identifying immunotherapy sensitivity subtypes in skin cutaneous melanoma. (A) UMAP visualization of 8132 SKCM cancer cells categorized into four clusters. (B) Kaplan–Meier curves showing PFS for 121 patients in SKCM discovery cohort. (C-D) Kaplan–Meier curves for PFS and OS in validation cohorts, including PFS-validation (*n* = 107) and OS-validation (*n* = 77). (E) Sankey diagram illustrating the association between immunotherapy response model predictions and pathological response states in discovery cohort. Fisher’s exact test was used to evaluate the association. (F) Kaplan–Meier curves of PFS for 65 SKCM patients receiving immunotherapy in pathological response group of discovery cohort. (G) Sankey diagram illustrating the association between immunotherapy response model predictions and pathological response states in PFS-validation cohort. (H) Kaplan–Meier curves of OS for 64 SKCM patients receiving immunotherapy in pathological response group of PFS-validation cohort. (I) Comparison of biomarker performance across different methods using C-index. (J) KEGG functional enrichment analysis of 167 genes distinguishing nonresponse and response groups in discovery cohort. (K) Split violin plots illustrating significant differences in infiltrating immune cell types between two response groups. (L) Split violin plots illustrating significant differences in immune stimulatory genes between two response groups. Statistical differences were calculated using Student’s t-test.

As a further extension of scBGDL, we focused on identifying predictive biomarkers for immunotherapy response in melanoma, a critical area of precision oncology. A total of 1019 prognostic genes were identified, and 8132 high-quality single cells (≥200 expressed genes) were retained (Fig. 5A). Integration of single-cell and bulk RNA-seq data enabled the development of a graph-based immunotherapy response model, which stratified patients into response and nonresponse groups based on a cut-off (0.7822). Survival analysis demonstrated that predicted nonresponders had significantly shorter PFS than responders (log-rank *P* = 1.10E-11, C-index = 0.6698, Fig. 5B). Validation in independent cohorts confirmed these results for both PFS (log-rank *P* = 2.36E-05, C-index = 0.6272, Fig. 5C) and OS (log-rank *P* = .0545, C-index = 0.5768, Fig. 5D) validation cohorts. Multivariate Cox analysis indicated that the model independently predicted survival after adjusting for clinical factors (Table S11). Furthermore, pathological response data from PFS discovery cohort (65 responders versus 56 nonresponders) highlighted its predictive value, as predicted nonresponders were significantly enriched in pathological nonresponse group (Fisher’s exact test, *P* = 2.10E-10, Fig. 5E). Among pathological responders, 30 were consistently predicted, while 35 were reclassified as nonresponders. Survival analysis showed that these reclassified nonresponders had significantly shorter PFS than consistent responders (log-rank *P* = 1.68E-05, C-index = 0.7065, Fig. 5F). Similar results were observed in PFS-validation cohort (64 responders versus 42 non responders), where 36 responders and 33 nonresponders were accurately classified, with significant enrichment (*P* = .0006, Fig. 5G). Reclassified nonresponders also showed significantly shorter PFS compared to consistently classified responders (log-rank *P* = .0125, C-index = 0.6299, Fig. 5H). We assessed scBGDL’s superiority in predicting immunotherapy response by comparing it with other methods and traditional clinicopathological models (Fig. 5I, Table S12). In both discovery and validation cohorts, scBGDL achieved the highest C-index values in most cases (0.7277 in PFS-discovery and 0.6141 in OS-validation cohorts), although the C-index in PFS-validation cohort (0.6283) was not the highest among the models. Notably, other methods, such as Scissor, scAB, and LP_SGL, also outperformed traditional clinical characteristics-based methods in discovery and validation cohorts, underscoring the advantage of integrating single-cell and bulk RNA-seq data for more accurate immunotherapy response predictions, supporting scBGDL as a valuable tool for personalized treatment strategies in melanoma.

Analysis of node-edge-group differences revealed a SKCM immunotherapy response network (Fig. S3A) with 24 high-risk and 60 low-risk core genes connected by 192 edges, and identified 14 key nonresponse driver edges (risk specificity >0.5; frequency >20%, Fig. S3B). Among the 19 node genes of the key driver edges, eight genes literature-validated associated with SKCM patients with immunotherapy (e.g. *TCF7, SP100, RUNX3,TNFRSF1B, SELPLG, and STAT4*). Functional analysis of 167 DEGs between response groups (FDR < 0.05) were significantly enriched in 41 KEGG functional pathways (Fig. 5J), covering 13 subcategories, which are directly relevant to immunotherapy. Predicted responders exhibited significantly higher abundances of 17 infiltrating immune cells types (Fig. 5K, Table S13), such as activated dendritic cells (*P* = 7.86E-06), activated B cells (*P* = 0.0054), Type 1 T Helper Cells (*P* = 0.0078), and activated CD8+ T cells (*P* = 0.0219). Additionally, 34 immune stimulatory and 11 HLA antigen-presentation genes were evaluated. Responders showed elevated expression levels of *CD28* [37] (*P* = .0329), *CD40* [38] (*P* = .0102), *TNF* [39] (*P* = .0002), *ICAM1* [40] (*P* = .0156), and *HMGB1* [41] (*P* = .0336) which enhance T-cell activation and overall immune response (Fig. 5L, Table S14). These results support the hypothesis that predicted responders are more likely to exhibit a positive response to immunotherapy, suggesting a promising approach for personalized treatment strategies.

## Discussion

The scBGDL offers a graph-based deep learning method for integrating single-cell RNA sequencing and bulk transcriptomic data, addressing critical challenges in the precise identification of cancer subtypes and clinical outcome prediction. By constructing sample-specific graph networks, scBGDL effectively captures the complexity of gene–gene interactions and cellular heterogeneity while mitigating the sparsity and noise often inherent in scRNA-seq data. The model employs state-of-the-art techniques, including GATs for feature aggregation, MinCutPool layers for dimensionality reduction, and Transformer modules to capture high-order interactions. These components collectively enable scBGDL to fully exploit multimodal transcriptomic data, ensuring both predictive accuracy and biological relevance. The validation of scBGDL across 16 diverse cancer types reveals its remarkable adaptability and robustness as a computational framework for diverse cancer types. While scBGDL consistently outperformed existing methods across most cancer types, the variability in performance metrics highlights the unique challenges posed by different cancer biology contexts. For example, the 16 cancer types selected for validation in this study encompass a diverse range of histological features and clinical presentations, including common epithelial cancers such as LUAD, CRC, and BRCA, as well as less common but highly aggressive malignancies like glioma and ESCA. This diverse cohort allows us to comprehensively evaluate scBGDL's performance across different cancer landscapes, thereby establishing its broad applicability in oncology research and clinical practice.

This method bridges the gap between fundamental research and clinical applications, showcasing versatility across cancer types. scBGDL was validated in three clinical therapeutic scenarios, highlighting its potential to improve cancer prognosis and treatment response. A key strength of scBGDL is its ability to enhance existing clinical indices. By integrating scBGDL-derived risk scores with traditional TNM staging into predictive nomograms, the model significantly improves prognostic accuracy across multiple cohorts, offering more precise patient outcome predictions compared to conventional indices: (i) scBGDL effectively stratified LUAD patients into high- and low-risk groups based on survival outcomes, achieving robust prognostic performance across multiple cohorts. Time-dependent AUCs were 0.68 (GSE68465), 0.59 (TCGA-LUAD), 0.78 (GSE31210), 0.73 (GSE30219), 0.72 (GSE50081), and 0.72 (GSE42127) (Fig. S4). Moreover, integrating scBGDL-derived prognostic scores with tumor stage and age in a predictive nomogram improved survival prediction efficiency, yielding higher C-index values than clinical nomograms alone in LUAD discovery (0.7414) and validation cohorts (TCGA-LUAD, 0.7338; GSE31210, 0.7781; GSE30219, 0.6920; GSE50081, 0.7087; GSE42127, and 0.7513; Table S15). These findings underscore the added value of scBGDL’s prognostic model. (ii) scBGDL accurately predicted EOC chemotherapy response, refining pathological classifications with AUCs of 0.76 (TCGA-OV), 0.62 (GSE9891), and 0.57 (GSE26193) (Fig. S5), supporting personalized treatment strategies. Integrating scBGDL-derived scores with tumor stage and grade into a predictive nomogram further improved prognostic precision, outperforming clinical features alone in TCGA-OV (0.7004) discovery and validation cohorts (GSE9891, 0.6594; GSE26193, 0.6896; Table S16), underscoring its potential for guiding personalized chemotherapy strategies in EOC. (iii) scBGDL predicted immune checkpoint blockade responses, identifying immune-related subtypes and therapeutic targets with AUCs of 0.88 (discovery), 0.67 (PFS-validation), and 0.63 (OS-validation) (Fig. S6), enabling tailored immunotherapy approaches.

This approach not only visualizes attention weights and key interactions but also elucidates the biological mechanisms underlying model decisions at the molecular interaction network level. In LUAD, we identified that postoperative recurrence is driven by a coordinated mitochondrial-metabolic-proteostasis network. The highest-weighted edge *CHCHD3*-*MRPL35* (Global Weight = 97.49) reflects *MRPL35*'s role in mitochondrial translation complex assembly [42]. The edge *CCDC90B*-*PYGL* (Global Weight = 45.22) aligns with reports of *PYGL*-mediated metabolic reprogramming [43], while *DPM1*-*PSMC4* (Global Weight = 15.55) corresponds to *PSMC4*-induced glycosylation defects and proteasomal inhibition [44]. These driver edges collectively demonstrate discriminative power for stratifying LUAD patients into high- and low-risk subgroups. In EOC, integrated analysis of attention weights and literature validation revealed a multidimensional paradigm of platinum resistance. The dominant edge *C8orf33*-*PROM2* (Global Weight = 143.47) mediates drug efflux through *PROM2* extracellular vesicles, depleting intracellular platinum in resistant tumors [45]. The *DMWD*-*SLC7A11* edge (Global Weight = 68.07) maintains redox homeostasis via glutathione synthesis [46]. Immune evasion is orchestrated by the *CMTM4*-*SOGA1* interaction (Global Weight = 87.16), wherein *CMTM4* stabilizes *PD-L1* and *SOGA1* promotes microenvironment acidification [47]. These edges robustly distinguish platinum nonresponders from responders in EOC patients receiving ACT. In SKCM, graph attention network analysis uncovered that immunotherapy resistance arises from cooperative failures in T-cell functionality, antigen presentation, and tissue residency. The attenuated *SELL-TCF7* interaction (Global Weight = 62.25) in nonresponders impairs T-cell differentiation [5]. Concurrently, ribosomal hijacking via the *RPS20-SURF2* edge (Global Weight = 68.97) is negatively associated with MHC-I antigen presentation; this suggests a hypothesis that ribosomal/translation dysregulation may affect antigen presentation, which requires targeted experimental validation [48]. Hyperactivation of the *SELPLG-SLC9A3R1* axis (Risk_Bias = 0.615) shortens immunological synapse duration, while disruption of the *RUNX3-SP100* interaction (Global Weight = 39.31) epigenetically silences tissue-residency genes through SP100-dependent chromatin remodeling [49, 50]. These driver edges provide a testable biological hypotheses for discriminating immunotherapy nonresponders in SKCM.

These findings not only validate scBGDL’s predictions but also provide actionable biological hypotheses for further exploration in cancer therapy. However, this study had some limitations. Its generalizability may be constrained by dataset heterogeneity, especially in smaller cohorts like those for SKCM immunotherapy. Larger, more diverse datasets are needed to improve validation. Additionally, batch effects and variability across scRNA-seq and bulk RNA-seq datasets could impact integration accuracy, necessitating advanced harmonization techniques. Future research will focus on deeper biological validation and the refinement of our model to address these limitations.

In conclusion, scBGDL establishes a novel graph-based deep learning framework that robustly integrates single-cell and bulk transcriptomic data. The consistent superiority of scBGDL in prognostic stratification across 16 diverse cancer types demonstrates its strong generalizability and potential for broad clinical application. The model's versatility is further evidenced by scenario-specific applications: LUAD survival prediction, EOC chemotherapy response assessment, SKCM immunotherapy outcome forecasting. By enhancing clinical indices and uncovering actionable biological insights, scBGDL bridges computational biology and clinical practice, providing a scalable computational paradigm for advancing precision oncology.

**Key Points**

- scBGDL is a graph-based deep learning method that integrates single-cell and bulk RNA-seq data, addressing challenges of data sparsity and noise to improve cancer subtype identification and prognosis prediction.
- Clinically versatile applications validated across multicenter cohorts: survival prediction in LUAD, chemotherapy response assessment in EOC, and immunotherapy outcome forecasting in SKCM.
- Comparative evaluations reveal scBGDL’s superior predictive accuracy, robustness, and scalability compared to existing methods.
- As an interpretable graph-based deep learning model, scBGDL effectively identifies critical pathways and molecular interactions relevant to cancer biology, and by providing transparent and biologically meaningful insights, it serves as a valuable tool for precision oncology.

## Supplementary Material

Supplemental_Materials_bbaf467

## Data Availability

The data sources and handling of the publicly available data used in this study are described in the Materials and Methods.

## Code availability

The source code for scBGDL model is available online (https://github.com/NEFLab/scBGDL).
