# Supplementary material for: Graph-based deep learning for integrating single-cell and bulk transcriptomic data to identify clinical cancer subtypes
Source: Brief Bioinform. 2025 Sep 11;26(5):bbaf467. doi: 10.1093/bib/bbaf467 (PMC12423395; doi:10.1093/bib/bbaf467)
Supplement: Supplemental_Materials_bbaf467 [file supplemental_materials_bbaf467.docx]

**SUPPLEMENTARY MATERIAL**

**Supplementary Methods**

**Molecular scores and immune cell infiltration**

The hypoxia score for a given sample was defined as the median expression of the 26 hypoxia genes [[1](#_ENREF_1)]. The hypoxia score was then normalized between 0 and 1, where 0 represented the least hypoxic and 1 represented the most hypoxic.

The proliferation score for a given sample was calculated by averaging the mRNA expression levels of 45 genes in the proliferation signature [[2](#_ENREF_2)]. A high proliferation score represented high proliferative capacity.

The stemness score for a given sample was calculated based on the mRNA of 109 stemness-related gene signatures by performing ssGSEA [[3](#_ENREF_3)] using a “GSVA” package. A high score represented high stemness of the tumours.

Seven cell death regulatory pathways of autophagy, apoptosis, pyroptosis, anoikis, immunogenic cell death, cuproptosis and ferroptosis were sourced from KEGG, REACTOME and literature.Based on the expression data of death regulator gene sets, death regulator scores were calculated by using a “GSVA” package

The immune score for a given sample was estimated by performing ssGSEA [[3](#_ENREF_3)], based on its mRNA expression profiles using an “estimate” package [[4](#_ENREF_4)].

**Supplementary Tables**

**Table S1. List of the datasets applied to develop scBGDL across 16 cancer types.**

| Cancer Type | RNA-seq Source | Patients | scRNA-seq Source | Patients | Cell |
| --- | --- | --- | --- | --- | --- |
| BLCA | TCGA-BLCA | 405 | BLCA_GSE130001 | 2 | 4,129 |
| Glioma | TCGA-GBM | 159 | Glioma_GSE163108 | 5 | 25,103 |
| BRCA | TCGA-BRCA | 1081 | BRCA_GSE176078 | 26 | 89,471 |
| CRC | TCGA-COAD | 424 | CRC_GSE166555 | 12 | 66,050 |
| ESCA | TCGA-ESCA | 183 | ESCA_GSE154763 | 7 | 7,673 |
| HNSC | TCGA-HNSC | 520 | HNSC_GSE180268 | 6 | 53,660 |
| KIRC | TCGA-KIRC | 531 | KIRC_GSE139555 | 3 | 49,907 |
| LIHC | TCGA-LIHC | 348 | LIHC_GSE140228 | 5 | 62,530 |
| NSCLC | TCGA-LUAD | 511 | NSCLC_E-MTAB-6149 | 5 | 40,218 |
| PAAD | TCGA-PAAD | 174 | PAAD_GSE154778 | 16 | 14,953 |
| CESC | TCGA-CESC | 291 | CESC_GSE168652 | 1 | 22,998 |
| OV | TCGA-OV | 377 | OV_GSE158722 | 21 | 96,846 |
| UCEC | TCGA-UCEC | 544 | UCEC_GSE139555 | 3 | 12,758 |
| PRAD | TCGA-PRAD | 496 | PRAD_GSE143791 | 16 | 36,850 |
| SKCM | TCGA-SKCM | 463 | SKCM_GSE159251 | 4 | 95,421 |
| STAD | TCGA-STAD | 386 | STAD_GSE167297 | 5 | 22,464 |

Note: BLCA, Bladder urothelial carcinoma; BRCA, Breast cancer; CRC, Colorectal cancer; ESCA, Esophageal Squamous Cell Carcinoma; HNSC, Head and neck squamous carcinoma; KIRC, Kidney Renal Clear Cell Carcinoma; LIHC, Liver hepatocellular carcinoma; NSCLC, Non-small cell lung cancer; PAAD, Pancreatic adenocarcinoma; CESC, Cervical Squamous Cell Carcinoma and Endocervical Adenocarcinoma; OV, Ovarian serous cystadenocarcinoma; UCEC, Uterine Corpus Endometrial Carcinoma; PRAD, Prostate Adenocarcinoma; SKCM, Skin Cutaneous Melanoma; STAD, Stomach adenocarcinoma.

**Table S2. Baseline clinical characteristics of LUAD patients treated with curative resection alone.**

|  | **E-MTAB-6149 scRNA datasets** | | | | | |
| --- | --- | --- | --- | --- | --- | --- |
| **Treatment** | Curative resection alone without adjuvant therapy | | | | | |
| **Sample** | 5 | | | | | |
| **Cells** | 40,218 | | | | | |
| **Platform** | 10x Genomics | | | | | |
|  | **Discovery** | **Bulk validation dataset** | | | | |
|  | **GSE68465** | **TCGA-LUAD** | **GSE31210** | **GSE50081** | **GSE30219** | **GSE42127** |
| **Sample** | *n* = 333 | *n* = 267 | *n* = 204 | *n* = 127 | *n* = 81 | *n* = 87 |
| **Age (years)** |  |  |  |  |  |  |
| **≤ 65** | 214(64.27%) | 152(56.93%) | 138(67.65%) | 76(59.84%) | 58(71.60%) | 51(58.62%) |
| **> 65** | 119(35.73%) | 115(43.07%) | 66(32.35%) | 51(40.16%) | 23(28.40%) | 36(41.38%) |
| **Gender** |  |  |  |  |  |  |
| **Female** | 111(33.33%) | 107(40.07%) | 82(40.20%) | 48(37.80%) | 36(44.44%) | 35(40.23%) |
| **Male** | 222(66.67%) | 160(59.93%) | 122(59.80%) | 79(62.20%) | 45(55.56%) | 52(59.77%) |
| **TNM stage** |  |  |  |  |  |  |
| **I** | 124 (37.24%) | 189 (70.79%) | 112(54.90%) | 68 (53.54%) | 72 (88.89%) | 45 (51.72%) |
| **II** | 145 (43.54%) | 45 (16.85%) | 92 (45.10%) | 59 (46.46%) | 9 (11.11%) | 32 (36.78%) |
| **III** | 34 (10.21%) | 20 (7.49%) | - | - | - | 10 (11.50%) |
| **IV** | 30 (9.01%) | 13 (4.87%) | - | - | - | - |
| **Average survival** * | 41.62 | 32.92 | 50.75 | 42.55 | 45.28 | 40.83 |
| **Platform** | Affy.U133A | Illu.HiSeq | Affy.Plus 2.0 | Affy.Plus 2.0 | Affy.Plus 2.0 | Affy.Plus 2.0 |

Note: * represents mean survival times after applying this 60-month threshold. Affy. Affymetrix; Illu. Illumina.

**Table S3. Baseline clinical characteristics of EOC patients treated with platinum-based ACT.**

|  | **GSE158722 scRNA dataset** | | |
| --- | --- | --- | --- |
| **Treatment** | Adjuvant Chemotherapy | | |
| **Sample** | 21 | | |
| **Cells** | 96,846 | | |
| **Platform** | 10x Genomics | | |
|  | **Bulk discovery dataset** | **Bulk validation dataset** | |
|  | **TCGA-OV** | **GSE9891** | **GSE26193** |
| **Sample** | *n* = 377 | *n* = 278 | *n* = 107 |
| **Age (years)** |  |  |  |
| **≤ 60** | 214 (56.76%) | 158 (56.83%) |  |
| **> 60** | 163 (43.24%) | 120 (43.17%) |  |
| **TNM stage** |  |  |  |
| **II** | 24 (6.37%) | 17 (6.12%) | 11 (10.28%) |
| **III** | 295 (78.25%) | 214 (76.98%) | 59 (55.14%) |
| **IV** | 54 (14.32%) | 22 (7.91%) | 17 (15.89%) |
| **Grade** |  |  |  |
| **Median** | 42 (11.14%) | 95 (34.17%) | 33 (30.84%) |
| **High** | 324 (85.94%) | 161 (57.91%) | 67 (62.62%) |
| **Residual tumor** |  |  |  |
| **0-10 mm** | 247 (65.52%) | 128(46.04%) |  |
| **≥ 11 mm** | 94 (24.93%) | 66 (23.74%) |  |
| **Histological subtype** |  |  |  |
| **Serous** | 377 (100%) | 260 (93.53%) | 79 (73.83%) |
| **Adenocarcinoma** |  | 1 (0.36%) | 3 (2.80%) |
| **Clear Cells** |  |  | 6 (5.61%) |
| **Endometrioid** |  | 17 (6.12%) | 7 (6.54%) |
| **Average survival** * | 35.57 | 31.84 | 36.01 |
| **Platform** | Illu.HiSeq | Affy.Plus 2.0 | Affy.Plus 2.0 |

Note: * represents mean survival times after applying this 60-month threshold. Affy. Affymetrix; Illu. Illumina.

**Table S4. Baseline clinical characteristics of SKCM patients receiving immunotherapy.**

|  | **GSE159251 scRNA datasets** | | | | |
| --- | --- | --- | --- | --- | --- |
| **Treatment** | Immunotherapy | | | | |
| **Sample** | 4 | | | | |
| **Cells** | 95,421 | | | | |
| **Platform** | 10x Genomics | | | | |
|  | **Bulk discovery** | **PFS validation** | | **OS validation** | |
|  | Liu’s study | Gide’s study | Van Allen’s study | Hugo’s study | Riaz’s study |
| **Sample** | *n* = 121 | *n* = 73 | *n* = 34 | *n* = 26 | *n* = 51 |
| **Age (years)** |  |  |  |  |  |
| **≤ 60** | - | 34(46.58%) | 19(55.88%) | 12(46.15%) | - |
| **> 60** | - | 39(53.42%) | 15(44.12%) | 14(53.85%) | - |
| **Gender** |  |  |  |  |  |
| **Female** | 50(41.32%) | 47(64.38%) | 13(38.24%) | 18(69.23%) | - |
| **Male** | 71(58.68%) | 26(35.62%) | 21(61.76%) | 8(30.77%) | - |
| **TNM stage** |  |  |  |  |  |
| **I** | 10(8.26%) | - | - | - | - |
| **II** | 7(5.79%) | - | - | - | - |
| **III** | 14(11.57%) | - | - | - | - |
| **IV** | 90(74.38%) | - | 34(100%) | 25(96.15%) | - |
| **TMB** | √ | √ | √ | √ | - |
| **ITH** | √ | - | - | - | - |
| **Tumor Purity** | √ | - | - | √ | - |
| **Average survival** * | 19.11 | 21.1 | 17.71 | 17.31 | 18.24 |
| **Platform** | Illu.HiSeq | Illu.HiSeq | Illu.HiSeq | Illu.HiSeq | Illu.HiSeq |

Note: TMB, Tumor Mutational Burden; ITH: Intra-Tumor Heterogeneity; * represents mean survival times after applying this 36-month threshold. Affy. Affymetrix; Illu. Illumina.

**Table S5. C-index model performances on survival prediction across 16 cancer types.**

| **Cancer_Type** | **scBGDL** | **Scissor** | **scAB** | **LP_SGL** | **vs. Scissor** | **vs. scAB** | **vs. LP_SGL** |
| --- | --- | --- | --- | --- | --- | --- | --- |
| TCGA-BLCA | 0.6914 | 0.513 | 0.6332 | 0.6513 | 34.82% | 9.19% | 6.16% |
| TCGA-GBM | 0.6676 | 0.5737 | 0.6593 | 0.6627 | 16.37% | 1.26% | 0.74% |
| TCGA-BRCA | 0.6809 | 0.6253 | 0.6855 | 0.6769 | 8.89% | -0.67% | 0.59% |
| TCGA-COAD | 0.6804 | 0.503 | 0.6794 | 0.6183 | 35.27% | 0.15% | 10.04% |
| TCGA-ESCA | 0.7909 | 0.5471 | 0.7215 | 0.7826 | 44.56% | 9.62% | 1.06% |
| TCGA-HNSC | 0.6086 | 0.5307 | 0.6042 | 0.6077 | 14.68% | 0.73% | 0.15% |
| TCGA-KIRC | 0.6714 | 0.5701 | 0.6651 | 0.6424 | 17.77% | 0.95% | 4.51% |
| TCGA-LIHC | 0.6872 | 0.5615 | 0.6388 | 0.6341 | 22.39% | 7.58% | 8.37% |
| TCGA-LUAD | 0.6511 | 0.6273 | 0.6137 | 0.6357 | 3.79% | 6.09% | 2.42% |
| TCGA-PAAD | 0.6803 | 0.6512 | 0.6709 | 0.6617 | 4.47% | 1.40% | 2.81% |
| TCGA-CESC | 0.8024 | 0.6221 | 0.7435 | 0.7993 | 28.99% | 7.92% | 0.39% |
| TCGA-OV | 0.6933 | 0.5300 | 0.6116 | 0.6007 | 30.81% | 13.36% | 15.42% |
| TCGA-UCEC | 0.7949 | 0.5677 | 0.7276 | 0.7266 | 40.02% | 9.25% | 9.40% |
| TCGA-PRAD | 0.8478 | 0.759 | 0.7779 | 0.7199 | 11.70% | 8.99% | 17.77% |
| TCGA-SKCM | 0.7114 | 0.5363 | 0.6712 | 0.6452 | 32.65% | 5.99% | 10.26% |
| TCGA-STAD | 0.6361 | 0.531 | 0.6304 | 0.6076 | 19.79% | 0.90% | 4.69% |
| Average | 0.7060 | 0.5781 | 0.6709 | 0.6670 | 22.12% | 5.23% | 5.85% |

**Table S6. Univariate and Multivariate Cox regression analysis in LUAD cohorts**

| **The GSE68465 cohort** | Univariate analysis | | Multivariate analysis | |
| --- | --- | --- | --- | --- |
|  | HR | *P* value | HR | *P* value |
| Signature (High risk vs. Low risk) | 3.04 | 2.16E-08 | 2.62 | 1.70E-06 |
| TNM stage (III vs. II vs. I) | 2.44 | 1.08E-16 | 2.15 | 2.00E-06 |
| Age (>65 vs. 65≤) | 1.03 | 0.0042 | 1.03 | 0.0030 |
| Sex (Male vs. Female) | 1.40 | 0.0625 | - | - |
| Lymphovascular invasion (Present vs. Absent) | 2.98 | 1.68E-09 | 1.08 | 0.7720 |
| **The GSE31210 cohort** | Univariate analysis | | Multivariate analysis | |
|  | HR | *P* value | HR | *P* value |
| Signature (High risk vs. Low risk) | 6.15 | 0.0008 | 4.61 | 0.0065 |
| TNM stage (III vs. II vs. I) | 4.07 | 0.0003 | 2.40 | 0.0306 |
| Age (>65 vs. 65≤) | 1.02 | 0.5595 | - | - |
| Sex (Male vs. Female) | 2.24 | 0.0438 | 1.89 | 0.1136 |
| **The GSE30219 cohort** | Univariate analysis | | Multivariate analysis | |
|  | HR | *P* value | HR | *P* value |
| Signature (High risk vs. Low risk) | 13.64 | 0.0102 | 13.64 | 0.0102 |
| TNM stage (III vs. II vs. I) | 1.40 | 0.4716 | - | - |
| Age (>65 vs. 65≤) | 1.03 | 0.1234 | - | - |
| Sex (Male vs. Female) | 1.21 | 0.6767 | - | - |
| **The GSE50081 cohort** | Univariate analysis | | Multivariate analysis | |
|  | HR | *P* value | HR | *P* value |
| Signature (High risk vs. Low risk) | 3.19 | 0.0001 | 3.02 | 0.0003 |
| TNM stage (III vs. II vs. I) | 2.19 | 0.0114 | 1.98 | 0.0285 |
| Age (>65 vs. 65≤) | 1.02 | 0.1376 | - | - |
| Sex (Male vs. Female) | 1.53 | 0.1694 | - | - |
| **The GSE42127 cohort** | Univariate analysis | | Multivariate analysis | |
|  | HR | *P* value | HR | *P* value |
| Signature (High risk vs. Low risk) | 3.21 | 0.0128 | 2.75 | 0.0320 |
| TNM stage (III vs. II vs. I) | 2.08 | 0.0149 | 1.69 | 0.0920 |
| Age (>65 vs. 65≤) | 1.05 | 0.0410 | 1.03 | 0.2130 |
| Sex (Male vs. Female) | 1.25 | 0.5839 | - | - |
| **The TCGA-LUAD cohort** | Univariate analysis | | Multivariate analysis | |
|  | HR | *P* value | HR | *P* value |
| Signature (High risk vs. Low risk) | 1.69 | 0.0287 | 1.47 | 0.1274 |
| TNM stage (III vs. II vs. I) | 2.55 | 7.70E-10 | 2.37 | 3.10E-08 |
| Age (>65 vs. 65≤) | 1.03 | 0.0072 | 1.03 | 0.0048 |
| Sex (Male vs. Female) | 1.15 | 0.5650 | - | - |

**Table S7. Comparison of the prediction performance of scBGDL against different methods and clinical features in the LUAD cohorts**

|  | scBGDL | Scissor | ScAB | LP_SGL | Stage | Age | Sex |
| --- | --- | --- | --- | --- | --- | --- | --- |
| GSE68465 | 0.6572 | 0.561 | 0.6514 | 0.6262 | 0.6715 | 0.5716 | 0.5425 |
| TCGA-LUAD | 0.5985 | 0.588 | 0.5357 | 0.5921 | 0.6822 | 0.5797 | 0.5415 |
| GSE31210 | 0.7458 | 0.729 | 0.6349 | 0.5809 | 0.6652 | 0.5595 | 0.6101 |
| GSE50081 | 0.6843 | 0.553 | 0.6090 | 0.6271 | 0.5852 | 0.5462 | 0.5409 |
| GSE30219 | 0.6565 | 0.597 | 0.6640 | 0.6181 | 0.5232 | 0.5766 | 0.5057 |
| GSE42127 | 0.7047 | 0.519 | 0.5894 | 0.6801 | 0.5868 | 0.6098 | 0.5356 |

**Table S8. Genetic lesions in 24 LUAD driver genes between high- and low-risk groups**

| **Entrez Gene ID** | **Symbol Gene ID** | **Genetic lesions** | ***P* value** | **Statistic** |
| --- | --- | --- | --- | --- |
| 143 | *PARP4* | Gene mutation | 0.0040 | -2.8989 |
| 324 | *APC* | Gene mutation/CNV deletion | 5.69E-08 | -5.5637 |
| 1130 | *LYST* | Gene mutation | 2.53E-12 | -7.2749 |
| 1499 | *CTNNB1* | Gene mutation | 0.5010 | -0.6731 |
| 1956 | *EGFR* | Gene mutation/CNV amplification | 3.54E-08 | -5.6460 |
| 3845 | *KRAS* | Gene mutation/CNV amplification | 0.0028 | 3.0112 |
| 4089 | *SMAD4* | Gene mutation/CNV deletion | 0.0002 | 3.7741 |
| 4233 | *MET* | Gene mutation/CNV amplification | 0.0317 | -2.1578 |
| 4763 | *NF1* | Gene mutation | 0.0118 | -2.5317 |
| 5290 | *PIK3CA* | Gene mutation | 4.83E-06 | 4.6487 |
| 5728 | *PTEN* | Gene mutation | 3.61E-05 | 4.1891 |
| 5921 | *RASA1* | Gene mutation | 0.2090 | 1.2601 |
| 5925 | *RB1* | Gene mutation | 0.0038 | 2.9120 |
| 6794 | *STK11* | Gene mutation | 7.13E-07 | -5.0554 |
| 7157 | *TP53* | Gene mutation | 0.7420 | 0.3298 |
| 7270 | *TTF1* | Gene mutation | 0.0084 | 2.6536 |
| 7750 | *ZMYM2* | Gene mutation | 0.9530 | 0.0591 |
| 8241 | *RBM10* | Gene mutation | 0.9510 | 0.0616 |
| 9612 | *NCOR2* | Gene mutation/CNV deletion | 0.0010 | -3.3197 |
| 9817 | *KEAP1* | Gene mutation | 0.0133 | -2.4922 |
| 23269 | *MGA* | Gene mutation/CNV deletion | 0.7610 | 0.3046 |
| 29072 | *SETD2* | Gene mutation/CNV deletion | 0.2930 | 1.0536 |
| 55193 | *PBRM1* | Gene mutation/CNV deletion | 9.24E-05 | -3.9587 |
| 55729 | *ATF7IP* | Gene mutation/CNV deletion | 0.9320 | -0.0852 |

**Table S9. Univariate and Multivariate Cox regression analysis in EOC cohorts**

| **The TCGA-OV cohort** | Univariate analysis | | Multivariate analysis | |
| --- | --- | --- | --- | --- |
|  | HR | *P* value | HR | *P* value |
| Signature (Non-response vs. Response) | 3.88 | 1.99E-19 | 3.57 | 3.60E-16 |
| Age (>60 vs. 60≤) | 1.37 | 0.0309 | 1.12 | 0.4690 |
| TNM stage (IV vs. III vs. II) | 1.17 | 0.3213 | - | - |
| Grade (High vs. Median) | 1.60 | 0.0581 | - | - |
| Residual tumor (0-10 mm vs. ≥ 11 mm) | 1.42 | 0.0329 | 1.49 | 0.0160 |
| **The GSE9891 cohort** | Univariate analysis | | Multivariate analysis | |
|  | HR | *P* value | HR | *P* value |
| Signature (Non-response vs. Response) | 1.80 | 0.0038 | 1.72 | 0.0079 |
| Age (>60 vs. 60≤) | 1.66 | 0.0101 | 1.79 | 0.0036 |
| TNM stage (IV vs. III vs. II) | 2.22 | 4.26E-05 | 2.16 | 0.0001 |
| Grade (High vs. Median) | 1.34 | 0.0895 | - | - |
| Residual tumor (0-10 mm vs. ≥ 11 mm) | 1.21 | 0.3844 | - | - |
| **The GSE26193 cohort** | Univariate analysis | | Multivariate analysis | |
|  | HR | *P* value | HR | *P* value |
| Signature (Non-response vs. Response) | 1.85 | 0.0136 | 2.00 | 0.0061 |
| TNM stage (IV vs. III vs. II) | 2.59 | 2.25E-05 | 1.99 | 1.20E-05 |
| Grade (High vs. Median) | 1.66 | 0.5766 | - | - |

**Table S10. Comparison of the prediction performance of scBGDL against different methods and clinical features in EOC cohorts**

|  | **scGDL** | **Scissor** | **ScAB** | **LP_SGL** | **Age** | **Stage** | **Grade** | **RS** |
| --- | --- | --- | --- | --- | --- | --- | --- | --- |
| TCGA-OV | 0.6933 | 0.5300 | 0.6116 | 0.6007 | 0.5511 | 0.5258 | 0.5321 | 0.5541 |
| GSE9891 | 0.6016 | 0.6130 | 0.5787 | 0.5497 | 0.5691 | 0.5994 | 0.5379 | 0.5413 |
| GSE26193 | 0.5779 | 0.5420 | 0.5407 | 0.5057 | - | 0.6349 | 0.5068 | - |

**Table S11. Univariate and Multivariate Cox regression analysis in the SKCM cohorts**

| **The discovery cohort** | Univariate analysis | | Multivariate analysis | |
| --- | --- | --- | --- | --- |
|  | HR | *P* value | HR | *P* value |
| Signature (Non-response vs. Response) | 10.30 | 1.01E-08 | 9.53 | 3.50e-08 |
| Tumor Mutational Burden | 1.00 | 0.3012 | - | - |
| Intra-Tumor Heterogeneity | 3.88 | 0.0488 | 2.09 | 0.2900 |
| TNM stage (III vs. II vs. I) | 0.97 | 0.9059 | - | - |
| Sex (Male vs. Female) | 0.93 | 0.4961 | - | - |
| Tumor Purity | 3.25 | 0.0150 | 1.92 | 0.1700 |
| **The PFS-validation cohort** | Univariate analysis | | Multivariate analysis | |
|  | HR | *P* value | HR | *P* value |
| Signature (Non-response vs. Response) | 2.91 | 4.98E-05 | 2.89 | 5.80E-05 |
| Age (>60 vs. 60≤) | 0.98 | 0.0276 | 0.98 | 0.0320 |
| Sex (Male vs. Female) | 0.90 | 0.6590 | - | - |
| **The OS-validation cohort** | Univariate analysis | | Multivariate analysis | |
|  | HR | *P* value | HR | *P* value |
| Signature (Non-response vs. Response) | 1.76 | 0.0578 | - | - |
| Tumor Mutational Burden | 1.00 | 0.2086 | - | - |
| Age (>60 vs. 60≤) | 1.04 | 0.1928 | - | - |
| TNM stage (III vs. II vs. I) | 1.89 | 0.9983 | - | - |
| Sex (Male vs. Female) | 1.08 | 0.9115 | - | - |
| Tumor Purity | 0.25 | 0.3391 | - | - |

**Table S12. Comparison of the prediction performance of scBGDL against different methods and clinical features in SKCM cohorts**

|  | Discovery | PFS validation | OS validation |
| --- | --- | --- | --- |
| ScBGDL | 0.7277 | 0.6283 | 0.6141 |
| Scissor | 0.61 | 0.642 | 0.552 |
| ScAB | 0.7071 | 0.6141 | 0.5459 |
| LP_SGL | 0.7033 | 0.5858 | 0.5529 |
| TMB | 0.5471 | - | 0.7093 |
| ITH | 0.5284 | - | - |
| Sex | 0.5007 | 0.5156 | 0.561 |
| Stage | 0.5051 | - | 0.532 |
| Purity | 0.6049 | - | 0.5581 |
| Age | - | 0.5782 | 0.5727 |

**Table S13. Differences in infiltrating immune cells types and tumor-killing immune cells between non-responders and responders in SKCM.**

| **Cells types** | ***P* value** | **Statistic** |
| --- | --- | --- |
| Activated B Cell | 0.0054 | -2.9486 |
| Activated CD4 T Cell | 0.0285 | -2.2385 |
| Activated CD8 T Cell | 0.0219 | -2.3896 |
| Activated Dendritic Cell | 7.86E-06 | -4.7832 |
| CD56bright Natural Killer Cell | 0.8120 | -0.2383 |
| CD56dim Natural Killer Cell | 0.0730 | -1.8228 |
| Central Memory CD4 T Cell | 0.1380 | -1.5036 |
| Central Memory CD8 T Cell | 0.0124 | -2.5590 |
| Effector Memory CD4 T Cell | 0.1580 | -1.4360 |
| Effector Memory CD8 T Cell | 0.0111 | -2.6557 |
| Eosinophil | 0.2270 | -1.2243 |
| Gamma Delta T Cell | 0.0047 | -2.9374 |
| Immature B Cell | 0.0002 | -4.1339 |
| Immature Dendritic Cell | 0.6830 | 0.4109 |
| Macrophage | 0.0030 | -3.1114 |
| Mast Cell | 0.0798 | -1.7817 |
| Myeloid-Derived Suppressor Cell | 0.0005 | -3.6876 |
| Memory B Cell | 9.91E-01 | -0.0113 |
| Monocyte | 0.0093 | -2.7032 |
| Natural Killer (NK) Cell | 0.0066 | -2.8271 |
| Natural Killer T (NKT) Cell | 0.0003 | -3.9259 |
| Neutrophil | 0.7190 | -0.3617 |
| Plasmacytoid Dendritic Cell | 0.0410 | -2.0974 |
| Regulatory T Cell (Treg) | 6.06E-05 | -4.2812 |
| T Follicular Helper (Tfh) Cell | 0.0030 | -3.0904 |
| Type 1 T Helper (Th1) Cell | 0.0078 | -2.7780 |
| Type 17 T Helper (Th17) Cell | 0.4140 | -0.8224 |
| Type 2 T Helper (Th2) Cell | 0.6080 | -0.5163 |

**Table S14. Immune modulatory gene differences between non-responders and responders in SKCM**

| **Gene symbol** | **Gene Role** | **Gene Function** | ***P* value** | **Statistic** |
| --- | --- | --- | --- | --- |
| *CD27* | Receptor | Stimulatory | 0.2720 | -1.1154 |
| *CD28* | Co-stimulator | Stimulatory | 0.0329 | -2.1837 |
| *CD40* | Receptor | Stimulatory | 0.0102 | -2.6337 |
| *CD80* | Co-stimulator | Stimulatory | 0.4460 | -0.7689 |
| *CX3CL1* | ligand | Stimulatory | 0.1980 | 1.3118 |
| *CXCL10* | ligand | Stimulatory | 0.0560 | -1.9443 |
| *CXCL9* | ligand | Stimulatory | 0.1390 | -1.5050 |
| *ENTPD1* | Other | Stimulatory | 0.0153 | -2.4638 |
| *HLA-A* | Antigen Presentation | N/A | 0.0020 | -3.1736 |
| *HLA-B* | Antigen Presentation | N/A | 0.0148 | -2.5203 |
| *HLA-C* | Antigen Presentation | N/A | 0.0208 | -2.3540 |
| *HLA-DPB1* | Antigen Presentation | N/A | 0.0005 | -3.5844 |
| *HLA-DQA1* | Antigen Presentation | N/A | 0.0119 | -2.5690 |
| *HLA-DQB1* | Antigen Presentation | N/A | 0.0099 | -2.6424 |
| *HLA-DQB2* | Antigen Presentation | N/A | 0.1690 | -1.3949 |
| *HLA-DRA* | Antigen Presentation | N/A | 0.0014 | -3.3466 |
| *HLA-DRB1* | Antigen Presentation | N/A | 0.0030 | -3.0406 |
| *HLA-DRB5* | Antigen Presentation | N/A | 0.0153 | -2.4819 |
| *HMGB1* | Other | Stimulatory | 0.0336 | -2.1498 |
| *ICAM1* | Cell adhesion | Stimulatory | 0.0156 | -2.4634 |
| *ICOS* | Receptor | Stimulatory | 0.4230 | -0.8099 |
| *ICOSLG* | Co-stimulator | Stimulatory | 0.7060 | -0.3792 |
| *IFNG* | ligand | Stimulatory | 0.3350 | -0.9752 |
| *IL12A* | ligand | Stimulatory | 0.7000 | 0.3875 |
| *IL1A* | ligand | Stimulatory | 0.6380 | 0.4732 |
| *IL1B* | ligand | Stimulatory | 0.9820 | 0.0227 |
| *IL2* | ligand | Stimulatory | 0.8810 | -0.1500 |
| *IL2RA* | Receptor | Stimulatory | 0.6440 | -0.4656 |
| *ITGB2* | Cell adhesion | Stimulatory | 4.11E-06 | -4.8601 |
| *MICB* | Antigen Presentation | N/A | 0.1980 | -1.3024 |
| *PRF1* | Other | Stimulatory | 0.0597 | -1.9290 |
| *TLR4* | Receptor | Stimulatory | 0.0385 | -2.1052 |
| *TNF* | ligand | Stimulatory | 0.0002 | -3.9199 |
| *TNFRSF14* | Receptor | Stimulatory | 0.0217 | -2.3319 |
| *TNFRSF18* | Receptor | Stimulatory | 0.4740 | -0.7222 |
| *TNFRSF9* | Receptor | Stimulatory | 0.4020 | -0.8457 |

**Table S15. Predictive performances of models for LUAD cohorts**

|  | **C-index (95% CIs)** | | | | | |
| --- | --- | --- | --- | --- | --- | --- |
|  | GSE68465 | TCGA-LUAD | GSE31210 | GSE30219 | GSE50081 | GSE42127 |
| Prognosis nomogram | 0.7414  (0.70–0.78) | 0.7338  (0.66–0.80) | 0.7781  (0.68–0.88) | 0.6920  (0.60–0.78) | 0.7087  (0.63–0.78) | 0.7513  (0.65–0.85) |
| GDL models | 0.6572  (0.61–0.70) | 0.5985  (0.52–0.68) | 0.7458  (0.65–0.84) | 0.6565  (0.56–0.75) | 0.6843  (0.64–0.66) | 0.7047  (0.59–0.82) |
| Clinical nomogram | 0.7058  (0.66–0.75) | 0.7195  (0.65–0.79) | 0.6906  (0.57–0.81) | 0.5834  (0.47–0.70) | 0.6248  (0.54–0.71) | 0.6558  (0.55–0.76) |

**Table S16. Predictive performances of models for EOC cohorts**

|  | **C-index (95% CIs)** | | |
| --- | --- | --- | --- |
|  | TCGA-OV | GSE9891 | GSE26193 |
| Predictive nomogram | 0.7004  (0.66–0.74) | 0.6594  (0.61–0.71) | 0.6896  (0.63–0.75) |
| GDL models | 0.6933  (0.65–0.73) | 0.6016  (0.55–0.66) | 0.5779  (0.59–0.72) |
| Clinical nomogram | 0.5417  (0.51–0.58) | 0.6038  (0.55–0.66) | 0.6549  (0.55–0.66) |

**Supplementary Figures**

**
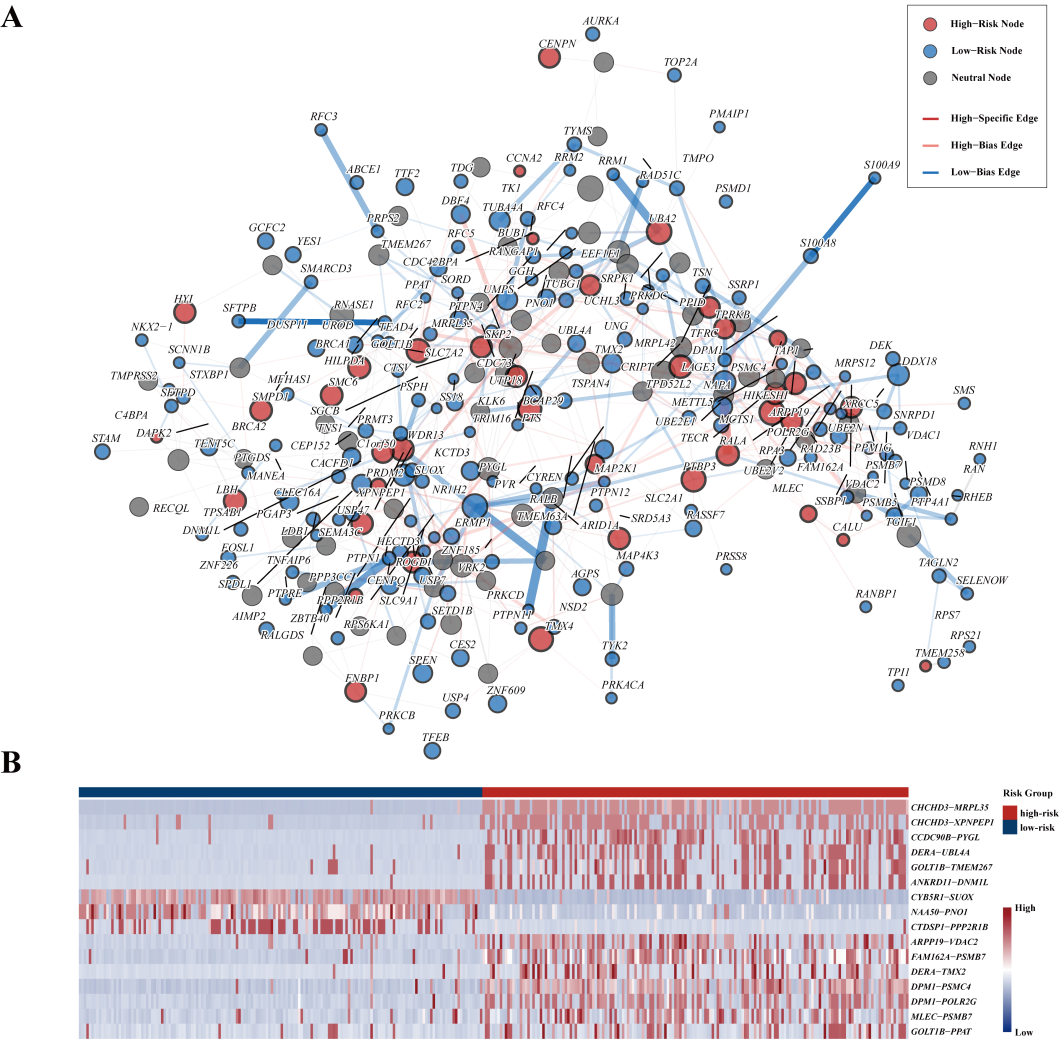
**

**Figure S1. Model interpretability and visualization for LUAD prognosis. (A)** Depicts the prognostic network associated with LUAD prognosis. Nodes represent genes (red for high-risk, blue for low-risk, gray for neutral), with size indicating importance. Edges show gene interactions (red for high-specificity, orange for high-bias, blue for low-bias) with size indicating importance. (B) This heatmap visualizes the weight levels of high-risk driver edges across high-risk and low-risk groups. Each row is a driver edge, each column a sample. Red indicates high expression, blue indicates low expression.

**
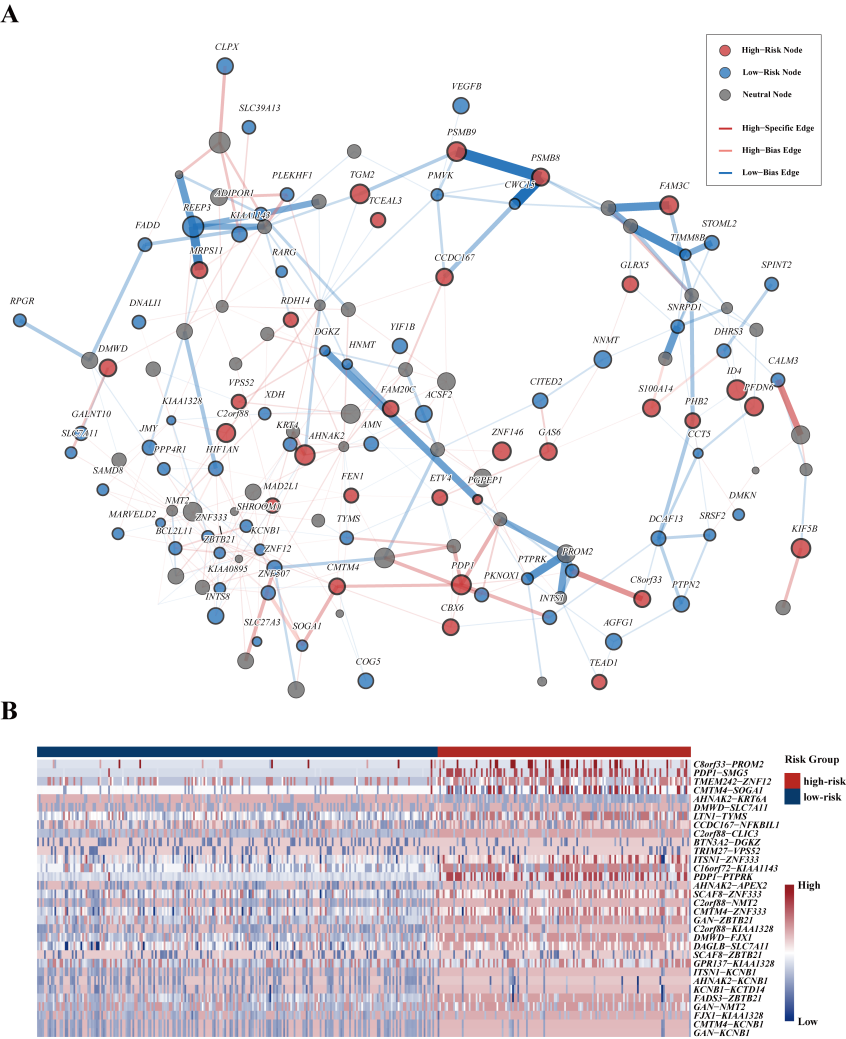
**

**Figure S2. Model interpretability and visualization for EOC platinum-based chemotherapy response. (A)** Depicts the prognostic network associated with EOC platinum-based chemotherapy response. Nodes represent genes (red for high-risk, blue for low-risk, gray for neutral), with size indicating importance. Edges show gene interactions (red for high-specificity, orange for high-bias, blue for low-bias) with size indicating importance. (B) This heatmap visualizes the weight levels of high-risk driver edges across high-risk and low-risk groups. Each row is a driver edge, each column a sample. Red indicates high expression, blue indicates low expression.

**
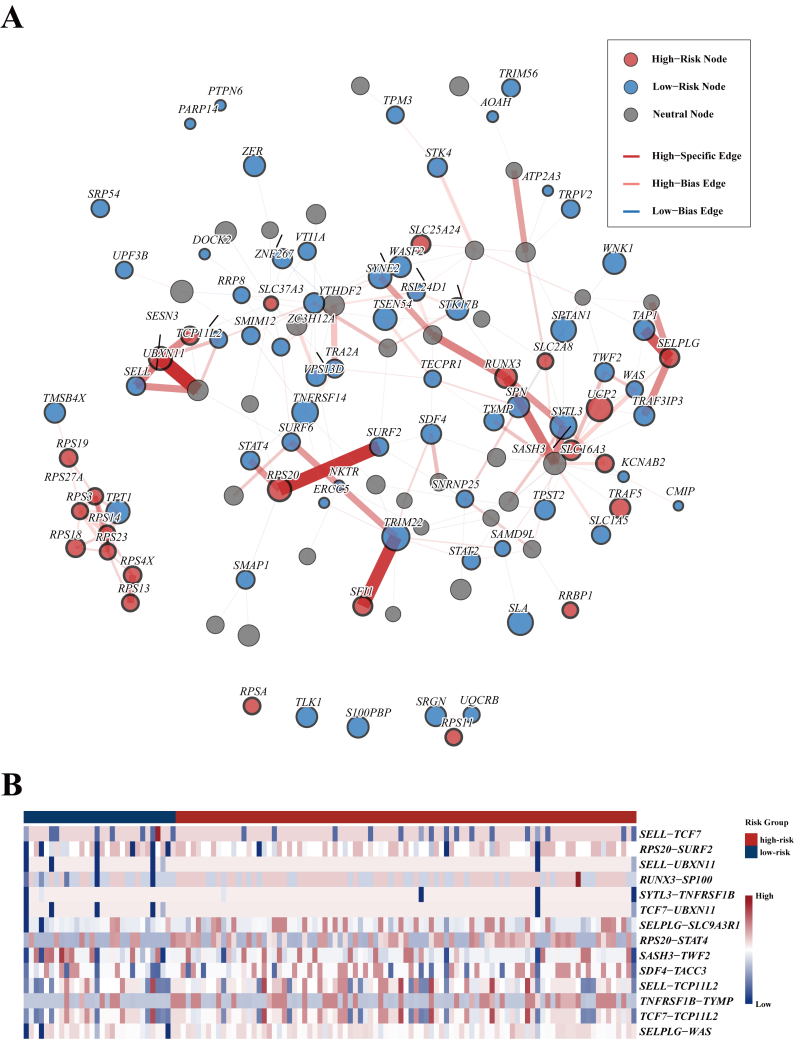
**

**Figure S3. Model interpretability and visualization for SKCM immunotherapy outcome. (A)** Depicts the prognostic network associated with SKCM immunotherapy outcome. Nodes represent genes (red for high-risk, blue for low-risk, gray for neutral), with size indicating importance. Edges show gene interactions (red for high-specificity, orange for high-bias, blue for low-bias) with size indicating importance. (B) This heatmap visualizes the weight levels of high-risk driver edges across high-risk and low-risk groups. Each row is a driver edge, each column a sample. Red indicates high expression, blue indicates low expression.


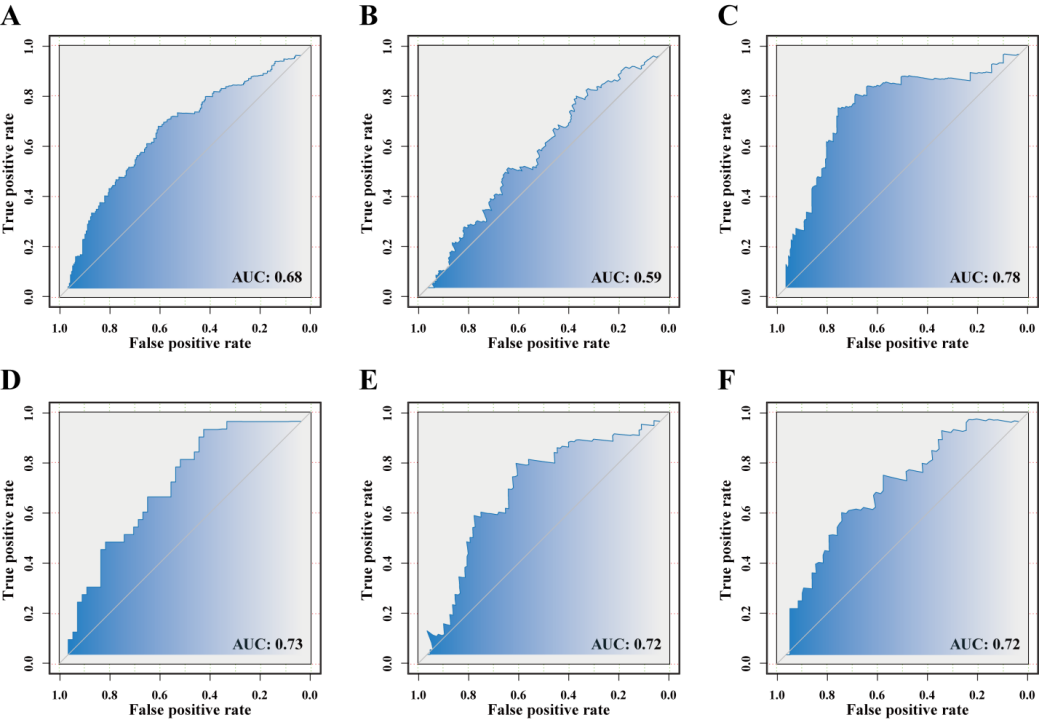


**Figure S4.** Time-dependent ROC curve of scBGDL in predicting 5-year survival rate in LUAD discovery and validation cohorts, including GSE68465 (*n*=333), TCGA-LUAD (*n*=267), GSE31210 (*n*=204), GSE50081 (*n*=127), GSE30219 (*n*=81), and GSE42127 (*n*=87).


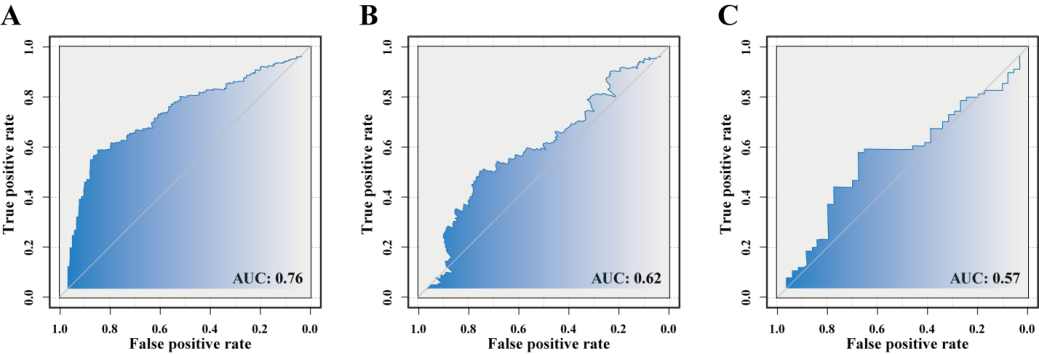


**Figure S5.** Time-dependent ROC curve of scBGDL in predicting 5-year survival rate in EOC discovery and validation cohorts, including TCGA-OV (*n*=377), GSE9891 (*n*=278) and GSE26193 (*n*=107).


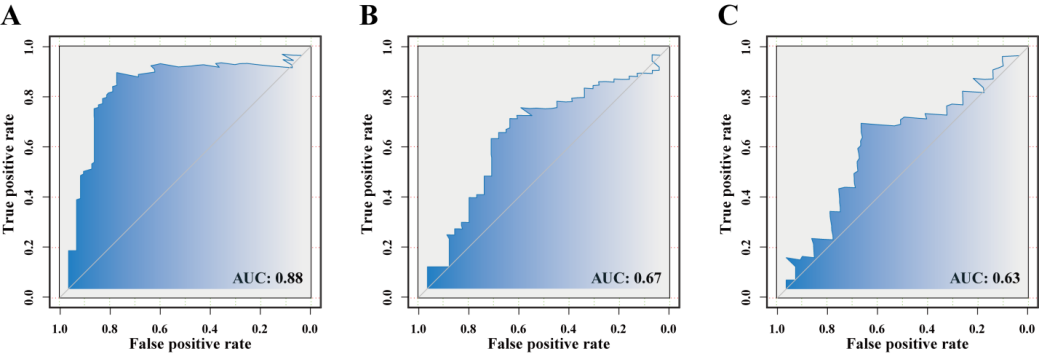


**Figure S6.** Time-dependent ROC curve of scBGDL in predicting 3-year survival rate in SKCM discovery and validation cohorts, including discovery (*n*=121), PFS-validation (*n*=107) and OS-validation (*n*=77).

**References for supplementary materials**

1. Eustace A, Mani N, Span PN et al. A 26-gene hypoxia signature predicts benefit from hypoxia-modifying therapy in laryngeal cancer but not bladder cancer, Clin Cancer Res 2013;19:4879-4888.

2. Whitfield ML, George LK, Grant GD et al. Common markers of proliferation, Nat Rev Cancer 2006;6:99-106.

3. Subramanian A, Tamayo P, Mootha VK et al. Gene set enrichment analysis: a knowledge-based approach for interpreting genome-wide expression profiles, Proc Natl Acad Sci U S A 2005;102:15545-15550.

4. Yoshihara K, Shahmoradgoli M, Martinez E et al. Inferring tumour purity and stromal and immune cell admixture from expression data, Nat Commun 2013;4:2612.
